# Calcium Pyruvate Exerts Beneficial Effects in an Experimental Model of Irritable Bowel Disease Induced by DCA in Rats

**DOI:** 10.3390/nu11010140

**Published:** 2019-01-10

**Authors:** Alba Rodríguez-Nogales, Francesca Algieri, Teresa Vezza, José Garrido-Mesa, José Alberto Molina-Tijeras, María Elena Rodríguez-Cabezas, María Pilar Utrilla, Ivo Pischel, Julio Gálvez

**Affiliations:** 1 CIBER-EHD, Department of Pharmacology, Center for Biomedical Research (CIBM), University of Granada, 18071 Granada, Spain; albarnogales@gmail.com (A.R.-N.); fra.algieri@hotmail.it (F.A.); teresavezza@hotmail.it (T.V.); josegarridomesa@gmail.com (J.G.-M.); jalbertomolinatijeras@gmail.com (J.A.M.-T.); merodri@ugr.es (M.E.R.-C.); utrillam@ugr.es (M.P.U.); 2 Instituto de Investigación Biosanitaria de Granada (ibs.GRANADA), 18012 Granada, Spain; 3 Dr. Ivo Pischel Consulting, 53547 Rossbach, Germany; ivopischel@aol.com; 4 Centre for Pharmacognosy and Phytotherapy, UCL School of Pharmacy, University of London, London WC1N 1AX, UK; 5 Department of Pharmacology, Center for Biomedical Research, University of Granada, Avenida del Conocimiento s/n 18016-Armilla, 18071 Granada, Spain

**Keywords:** calcium pyruvate, irritable bowel disease, deoxycholic acid, colorectal distension, referred pain

## Abstract

Pyruvate is a normal constituent of the body that participates in carbohydrate metabolism and functions as a scavenger of free radicals. Calcium pyruvate monohydrate (CPM) is a more stable derivative that has proved its anti-inflammatory effect in experimental colitis, among other disorders, and that could also be considered a source of calcium. Thus, it would be useful for the treatment of diseases with an inflammatory component and a high prevalence of osteoporosis like the irritable bowel syndrome (IBS). The aim of the present study is to evaluate the effects of CPM in a rat model of chronic post-inflammatory visceral pain induced by deoxycholic acid (DCA) that resembles IBS. Rats were administered DCA for three days intracolonically and then treated daily with CPM (40 and 100 mg/kg) or gabapentin (70 mg/kg) (positive control) by oral gavage for 17 days. The treatments reduced the visceral hypersensitivity measured by response to colorectal distension and referred pain. DCA induced changes in the colonic immune response characterized by increased expression of the cytokine *Il-1β* and the inducible enzyme *Cox-2*, which was reduced by the treatments. DCA also decreased the gut expression of the mucins *Muc-2* and *Muc-3*, which was normalized by CPM, whereas gabapentin only increased significantly *Muc-3*. Moreover, DCA increased the expression of *Tlr3*, which was decreased to basal levels by all the treatments. However, the serotonin receptor *Htr-4*, which was also elevated, was not affected by any of the treatments, indicating no effect through this signalling pathway. In conclusion, CPM ameliorated the visceral hypersensitivity and the referred pain caused by DCA, being as effective as the control drug. Furthermore, it improved the immune status of the animals, which could contribute to the visceral analgesia and the regeneration of the intestinal epithelial barrier integrity.

## 1. Introduction

Pyruvate, the anionic form of pyruvic acid (2-oxo-propanoic acid), is a pivotal biochemical intermediate of the carbohydrate metabolism, which, apart from being an important energy-bearing metabolite, acts as an endogenous scavenger of reactive oxygen species (ROS). The latter could explain its reported beneficial effects, including the improvement of the cardiac function after coronary ischemia and reperfusion and in critical medical conditions, like severe sepsis, acute respiratory distress syndrome, burn injury, acute pancreatitis, and stroke [1]. However, despite its potential benefits, pyruvate cannot be used in clinical practice due to its instability in solution [1]. Therefore, and in order to solve this problem, different pyruvate derivatives have been developed like calcium pyruvate monohydrate (CPM) that has been synthesized avoiding destabilizing reaction conditions [2]. CPM has been used to control obesity [3] and its capacity to lower the risk of hypertension and colon cancer has been also described [4]. Moreover, CPM has been reported to exert intestinal anti-inflammatory effects in an experimental model of colitis in rats [5]. Additionally, CPM, as a source of calcium, could be used to prevent osteoporosis [3]. Additionally, calcium pyruvate can be used as a safe source of calcium in food supplements with good bioavailability [3], while the corresponding intake of pyruvate is not of safety concern as it is a normal constituent of the body that enters the Krebs cycle or anaerobic metabolism.

Taking into account the different properties described for CPM, scavenger of ROS, anti-inflammatory and prevention of osteoporosis, we consider that CPM can be a new strategy to treat diseases with an immune component in which there is also a dysregulation in the calcium homeostasis. This may be the case of irritable bowel syndrome (IBS), since several studies have reported a lower calcium intake in IBS patients compared to general population [6,7,8]. According to Rome IV criteria, IBS is a chronic functional bowel disorder characterized by recurrent abdominal pain associated with defecation, or with a change in the texture of stools, or with a change in the frequency of stooling. Furthermore, it has also been reported an increased risk of osteoporotic fractures in IBS patients, even higher than in inflammatory bowel disease patients [9]. Currently, the particular mechanisms involved in the loss of bone mineral density in these patients are unknown. Nevertheless, it has been proposed a reduced intake of calcium since many patients avoid the consumption of dairy products because they have or may think they have lactose intolerance [10,11]. In addition, other factors related to its pathophysiology, such as gut mucosa inflammation and permeability changes could be implicated. Several studies have shown an altered immune response in IBS patients characterized by increased activity of T- and B-lymphocytes, and thus elevated levels of circulating pro-inflammatory cytokines such as IL-1β, TNF-α, IL-6, and IL-8 [12,13], which could promote bone resorption and osteoporosis [14]. Moreover, mast cells, which are located close to nerve fibres, could contribute to the development of chronic visceral hyperalgesia and more intense abdominal pain [15,16] which has also been described to lead to a reduced absorption of vitamin D and calcium that can as well account for the increased risk of osteopenia and osteoporosis [17].

Although a wide range of novel pharmacological compounds improve visceral symptoms of IBS, there is no established treatments for bone loss in IBS, maybe because its underlying pathophysiology is poorly understood [18]. Thus, the search for effective IBS therapeutic strategies remains a significant clinical challenge. Therefore, the aim of the present study was to evaluate the potential use of stable and pure CPM in the treatment of a model of post-inflammatory IBS, which has previously reported to exert intestinal anti-inflammatory effects in acute and chronic murine models of colitis, and to be a form of calcium supplementation for the treatment of osteoporosis in postmenopausal women with good bioavailability and tolerability [5,19]. With this purpose, the effects of CPM have been evaluated in a rat model of chronic post-inflammatory visceral pain induced by deoxycholic acid (DCA) that results in persistent visceral hyperalgesia and referred pain in rats, modelling some aspects of post-inflammatory IBS in humans [20].

## 2. Materials and Methods

### 2.1. Chemicals and Reagents

Calcium pyruvate monohydrate (CPM) was provided by PhytoLab GmbH and Co. KG (Vestenbergsgreuth, Germany). All other chemicals were obtained from Sigma–Aldrich Quimica (Madrid, Spain), unless otherwise stated.

### 2.2. Rat Model of Chronic Post-Inflammatory Visceral Pain Induced by DCA

Adult male Sprague Dawley rats (240–320 g) from Janvier Labs (St Berthevin Cedex, France) were housed in makrolon cages, maintained in an air-conditioned atmosphere with a 12 h light–dark cycle, and provided with food and tap water ad libitum. This study was carried out in accordance with the “Guide for the Care and Use of Laboratory Animals” as promulgated by the National Institute of Health and the protocols approved by the Ethic Committee of Laboratory Animals of the University of Granada (Granada, Spain) (ref. no. CEEA-2010-286). All studies involving animals are reported in accordance with the ARRIVE guidelines for reporting experiments involving animals [21,22]. The rats were randomly assigned to five groups (*n* = 8). Two groups were treated with CPM (40 and 100 mg/kg) and other with gabapentin (70 mg/kg), doses chosen by preliminary studies. All compounds were dissolved in 1 mL of carboxymethylcellulose (0.2%) in water solution, and administered daily by oral gavage. An untreated DCA control group and a saline group were included for reference, which were given the vehicle used to administer the test compounds. Rats were fasted overnight, anesthetized with halothane and a gavage needle was inserted through the anus approximately 6 cm into the colon and 1 mL of 4 mmol/L DCA in Kreb’s solution (in mmoles: NaCl, 122; KCl, 3.5; NaHCO_3_, 25; KH_2_PO_4_, 1.2; MgCl_2_, 1.2; pH 7.4) was injected while the needle was slowly withdrawn. Rats were left on a mound of bedding in a head-down position to prevent leakage of DCA. Rats were injected once daily for three consecutive days, the first injection counting as day 1. Rats in the control group received 1 mL 0.9% saline instead of DCA. The treatments were given from the last day of the DCA injection until the sacrifice of the rats with an overdose of halothane, 17 days later. Animal body weights, occurrence of diarrhea, and water and food intake were recorded daily throughout the experiment. Once the animals were sacrificed, the colon was removed aseptically and placed on an ice-cold plate, longitudinally opened and cleaned from their luminal contents with cold saline. The colon was subsequently minced, aliquoted and kept frozen at −80 °C until biochemical determinations and RNA or protein extraction were performed.

For the biochemical evaluation of tissue damage, colonic myeloperoxidase (MPO) activity was evaluated following the technique reported by Krawisz et al. (1984) [23], and the results were expressed as MPO units per gram of wet tissue; one unit of MPO activity was defined as that degrading 1 μmol hydrogen peroxide/min at 25 °C.

For the analysis of gene expression in colonic samples by RT-qPCR, total RNA from colonic samples was isolated using Trizol^®^ (Thermo Fisher Scientific Inc., Waltham, MA, USA) following the manufacturer’s protocol. All RNA samples were quantified with the Thermo Scientific NanoDrop™ 2000 Spectrophotometer (Thermo Fisher Scientific Inc., Waltham, MA USA) and 2 μg of RNA were reverse transcribed using oligo (dT) primers (Promega, Southampton, UK). Real time quantitative PCR amplification and detection was performed on optical-grade 48 well plates in an Eco™ Real-Time PCR System (Illumina, San Diego, CA, USA) with 20 ng of cDNA, the KAPA SYBR^®^ FAST qPCR Master Mix (Kapa Biosystems, INC, Wilmington, MA, USA) and specific primers at their annealing temperature (Ta) (Table 1). To normalize mRNA expression, the expression of the housekeeping gene, glyceraldehyde-3-phosphate dehydrogenase (*Gapdh*) was measured. The mRNA relative quantitation was calculated using the ΔΔCt method.

Some colonic samples (*n* = 6) were processed as described previously to evaluate COX-2 protein expression by Western blotting [24]. Equal amounts of protein from tissue samples (150 μg) were separated on 10% SDS-PAGE and transferred to a PVDF membrane. The membrane was then incubated with the antibodies anti-COX2 (Cell Signaling Technology, Danvers, MA, USA) and anti-β-actin used as loading control. Peroxidase-conjugated anti-rabbit IgG and anti-mouse IgG were used as secondary antibodies, respectively. Then, ECL (Perkin Elmer^TM^, Life Sciences, Boston, MA, USA) detection was performed. The quantification of bands was performed by densitometric analysis using ImageJ software (Free Software Foundation Inc., Boston, MA, USA).

### 2.3. Measurement of Response to Colorectal Distension

Visceral hypersensitivity was measured seven and fourteen days after the last injection of DCA by the response of rats to colorectal distension (CRD). First of all, rats were weakly sedated with isoflurane. Then a latex balloon catheter was inserted 5 cm into the colon and fixed to the base of the tail. Rats were placed in small plastic cages and allowed to adapt for 30 min. CRD was performed by inflating the balloon to a constant pressure measured with a sphygmomanometer connected to a pressure transducer. The balloon was inflated to 60 mmHg, for a 20 s stimulation period followed by a 5 min rest. Behavioural responses to CRD were measured by visual observation of the abdominal withdrawal reflex (AWR) by a blinded observer and the assignments of an AWR score were as follows: 0 = Normal behaviour without response; 1 = brief head movement at the onset of the stimulus followed by immobility; 2 = contraction of abdominal muscles; 3 = lifting of the abdomen off the platform; 4 = body arching and lifting of pelvic structures [25,26].

### 2.4. Determination of Referred Pain

Animals were tested for referred hyperalgesia seven and 14 days after the last injection of DCA. The lower back was shaved, rats were placed in individual plastic boxes, and after acclimation a series of von Frey filaments (Stoelting Co, Wood Dale, IL, USA) ranging from 8 g down to 1 g were applied perpendicularly to the lower back. A brisk escape was considered a positive response. Each filament was tested 5 times for 10 s. If the rat had a positive response (at least one escape response), the filament of next lower force was applied until two filaments were tested without a positive response.

### 2.5. In Vivo Intestinal Permeability

Some rats from the different experimental groups (*n* = 4) were fasted for 12 h and given DX-4000–FITC by oral gavage (350 mg/kg body weight). After 4 h, blood was collected from the abdominal aorta and centrifuged at 4 °C, 3000 rpm for 10 min. Plasma was diluted (1:20) in PBS (pH 7.4) and analysed for DX-4000–FITC concentration with a fluorescence spectrophotometer (Fluorostart, BMG Labtechnologies, Offenburg, Germany) at an excitation wavelength of 485 nm and emission wavelength of 535 nm. Standard curves were obtained by diluting FITC–dextran in PBS [27].

### 2.6. Statistics

All results are expressed as the mean ± standard error of the mean (SEM). Differences between means were tested for statistical significance using a one-way analysis of variance (ANOVA) and post hoc least significance tests. Non-parametric data were analysed by the Kruskal–Wallis test. The von Frey data were reported as area under the curve. All statistical analyses were carried out with the GraphPad Prism version 7 (GraphPad Software Inc., La Jolla, CA, USA), with statistical significance set at *p* < 0.05.

## 3. Results and Discussion

**Figure 1:**
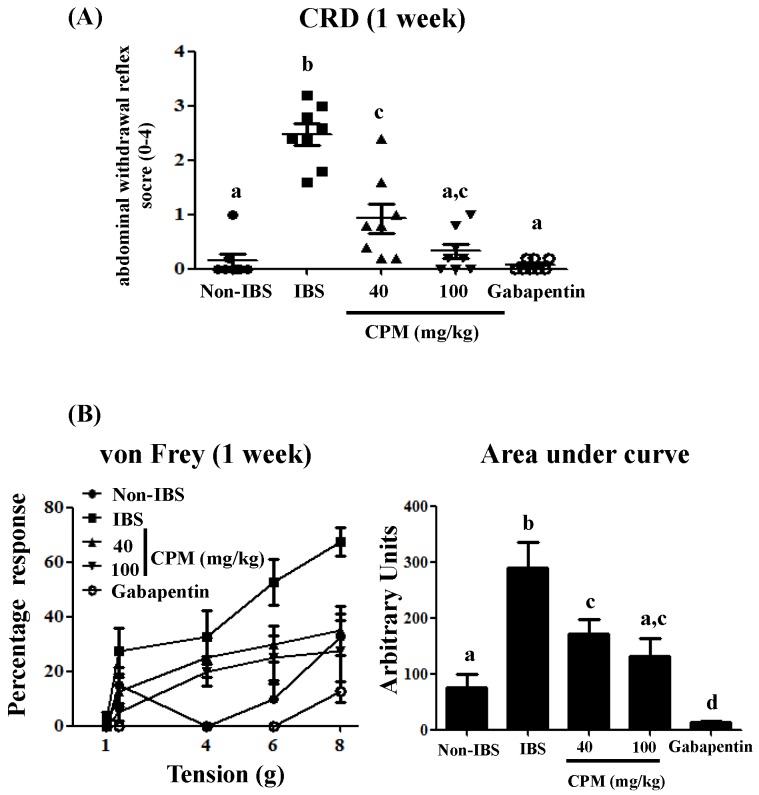
(**A**) Response to colorectal distension (CRD) of 60 mm Hg with a balloon catheter (duration = 20 s, interstimulus interval = 5 min) one week after DCA intracolonic administration in rats treated with calcium pyruvate monohydrate (CPM) (40 mg/kg and 100 mg/kg) or gabapentin (70 mg/kg) (*n* = 8). (**B**) Evaluation of referred hyperalgesia one week after DCA administration induced by von Frey filaments (1–8 g) applied to the lower back (5 times for 10 s) in rats treated with CPM (40 mg/kg and 100 mg/kg) or Gabapentin (70 mg/kg). Referred hyperalgesia was measured by percentage of response to von Frey filaments and the area under the curve (AUC) was calculated. Data are expressed as means ± SEM (*n* = 8). Groups with different letters statistically differ (*p* < 0.05).

**Figure 2:**
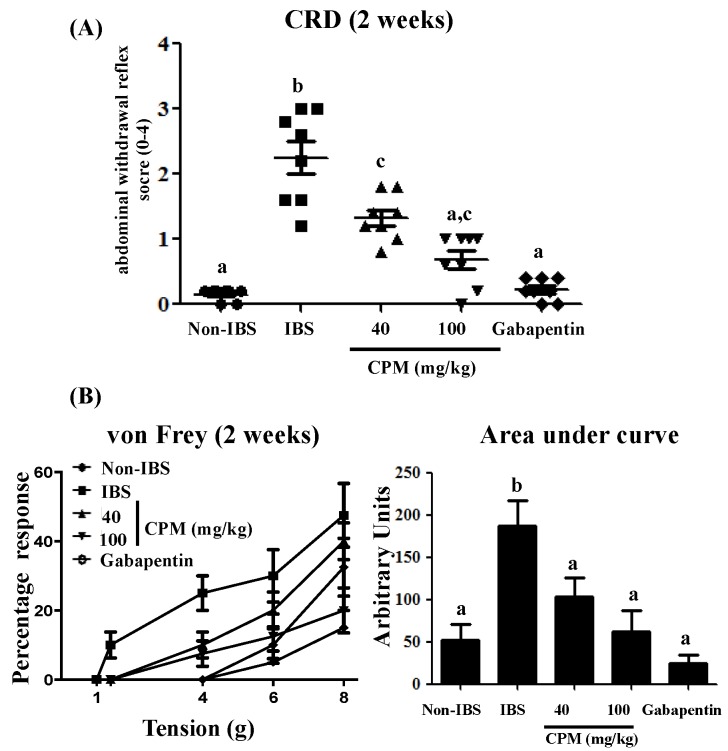
(**A**) Response to colorectal distension (CRD) of 60 mmHg with a balloon catheter (duration = 20 s, interstimulus interval = 5 min) two weeks after DCA intracolonic administration in rats treated with calcium pyruvate monohydrate (CPM) (40 mg/kg and 100 mg/kg) or gabapentin (70 mg/kg). (**B**) Evaluation of referred hyperalgesia two weeks after DCA administration induced by von Frey filaments (1–8 g) applied to the lower back (five times for 10 s) in rats treated with CPM (40 mg/kg and 100 mg/kg) or Gabapentin (70 mg/kg). Referred hyperalgesia was measured by percentage of response to von Frey filaments and the area under the curve (AUC) was calculated. Data are expressed as means ± SEM (*n* = 8). Groups with different letters statistically differ (*p* < 0.05).

Previous studies have shown that the repetitive intracolonic instillation of DCA (4 mM) to rats induces a mild and transient colonic inflammation, with no signs of ulceration or epithelial damage, characterized by persistent visceral hyperalgesia for at least four weeks [20]. In the present study, the administration of DCA for three consecutive days resulted in increased visceral hypersensitivity to colorectal distension (CRD) (60 mm Hg) one and two weeks after, in comparison with the saline group (Figure 1 and Figure 2). However, the oral administration of CPM, at doses of 40 and 100 mg/kg, reduced the CRD scores dose-dependently at both time points evaluated when compared with the IBS control group (Figure 1 and Figure 2). Of note, at the highest dose of CPM assayed, no significant differences were observed in CRD scores in comparison with gabapentin, which was also able to reduce the visceral hyperalgesia to similar score values to those obtained for the saline group (Figure 1 and Figure 2).

When the referred pain was examined by the response produced in the lower abdomen with the von Frey filaments, it was observed that visceral hyperalgesia in DCA-treated rats was associated with secondary hyperalgesia that resulted in a facilitated response of escape after the mechanical stimulation of non-injured tissue. Thus, rats from untreated DCA control group showed an elevated sensitivity since the threshold for the response was lower than in the saline group, with significant increases in the percentages of responses at all the pressures assayed at both time points evaluated, being more intense one week after DCA instillation than two weeks after (Figure 1 and Figure 2). The administration of CPM, at doses of 40 and 100 mg/kg, significantly reduced the percentage of response at the different pressures assayed in comparison with the DCA control group (Figure 1 and Figure 2). The AUC values were normalized with CPM (100 mg/kg) from the first week, and at the second week for the lowest dose, showing similar values to the saline control group and the gabapentin treated group (Figure 1 and Figure 2). Therefore, both treatments, CPM and gabapentin, significantly improved the increased sensitivity induced by DCA instillation. This shows the potential beneficial effects that CPM may exert in IBS, which are similar to those displayed by gabapentin, a GABAergic agent that has been clinically assayed for visceral pain in human IBS [28].

**Figure 3:**
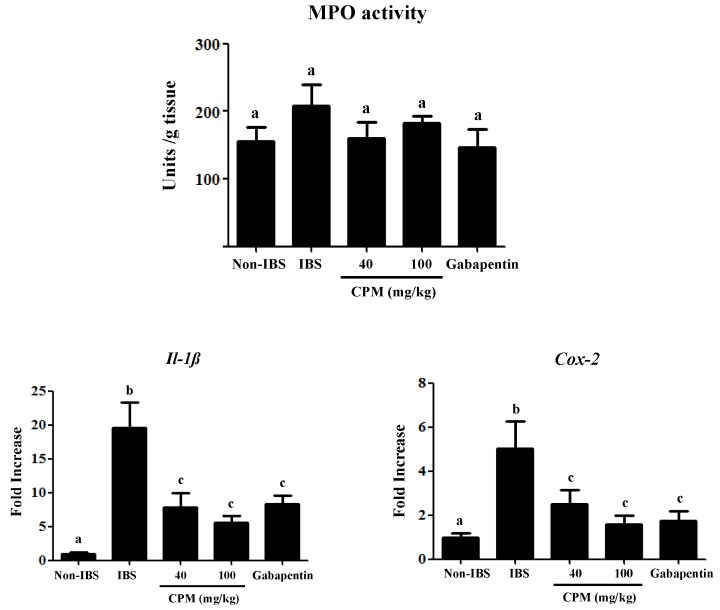
Effects of CPM (40 mg/kg and 100 mg/kg) and gabapentin (70 mg/kg) on colonic myelopereoxidase (MPO) activity and gene expression of *Il-1β* and *Cox-2* in rats two weeks after DCA administration. Data are expressed as means ± SEM (*n* = 8). Groups with different letters differ statistically (*p* < 0.05).

**Figure 4:**
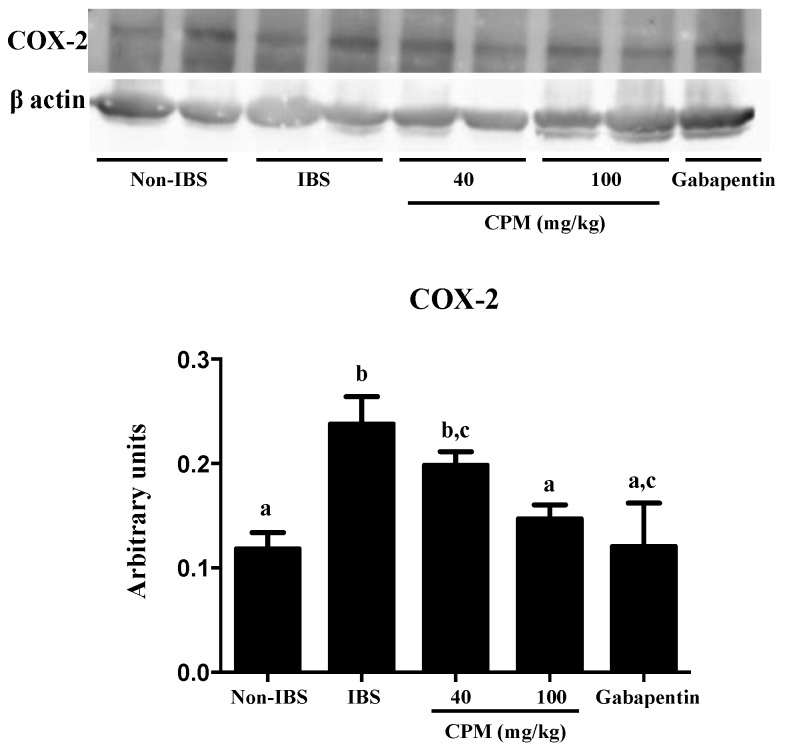
Effects of CPM (40 mg/kg and 100 mg/kg) and gabapentin (70 mg/kg) on COX-2 protein expression (Western blot) in rats two weeks after DCA administration. Experiments were performed in triplicate. The quantification of bands was performed by densitometric analysis using ImageJ software. Data are expressed as means ± SEM (*n* = 6). Groups with different letters differ statistically (*p* < 0.05).

**Figure 5:**
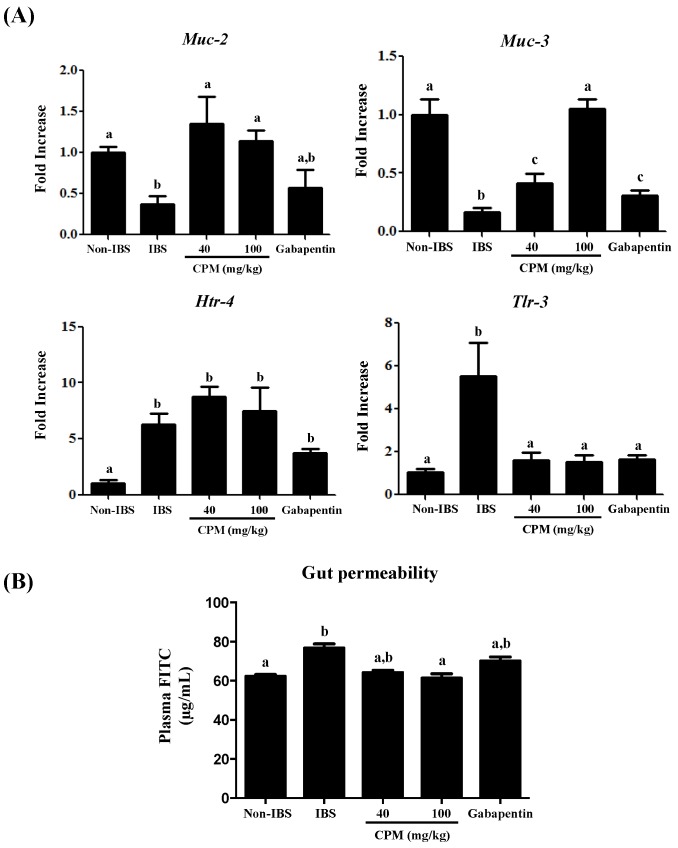
Effects of CPM (40 mg/kg and 100 mg/kg) and gabapentin (70 mg/kg) on: (**A**) colonic gene expression of *Muc-2*, *Muc-3, Htr-4*, and *Tlr-3*, and (**B**) intestinal permeability measurement by the FITC-dextran assay in vivo in rats two weeks after DCA administration. Data are expressed as means ± SEM (*n* = 8 for colonic gene expression and *n* = 4 for intestinal permeability). Groups with different letters differ statistically (*p* < 0.05).

The biochemical analysis of the colon after two weeks revealed an almost complete recovery from the initial DCA-induced inflammatory damage. Thus, no differences were found among different experimental groups in the colonic MPO activity (Figure 3), a common marker of neutrophil infiltration in intestinal inflammatory conditions [23]. This agrees with previous studies in the same experimental model that reported a mild, short-lasting inflammatory response in the colon [20]. Interestingly, patients with IBS also show a much lower immune infiltrate than those with active or quiescent ulcerative colitis [29]. However, at this time point, an altered immune response was still evident since the expressions of the cytokine *Il-1β* and the inducible enzyme *Cox-2* were increased in the untreated control IBS group in comparison with the saline control (Figure 3), as described in human IBS and in other experimental models [30,31]. Although the inflammatory component in IBS is controversial [32], it has been described that the immune system activation is implicated in the pathophysiology of IBS [12], in which stimulated mast cells seem to play a key role due to the release of different biologically active substances, including not only histamine, serotonin and proteases, but also cytokines, like *Il-1β*, and membrane-derived arachidonic acid metabolites like prostaglandins, the latter derived from the increased expression of the enzyme *Cox-2* [30]. The administration of CPM, at both doses assayed, significantly reduced colonic *Cox-2* expression, similarly to the effects obtained with gabapentin (Figure 3). The effect of the different treatments on *Cox-2* expression was corroborated when its colonic protein levels were evaluated by Western blot (Figure 4). The reduced expression of colonic *Cox-2* in this experimental model of IBS could result in a decrease in prostaglandin production and secretion. This may ameliorate the hyperalgesia typically ascribed to these eicosanoids, given their ability to reduce the activation threshold of sensory afferents through the activation of the corresponding receptors EP1 [33]. Furthermore, the treatments significantly reduced the colonic expression of *Il-1β* (Figure 3), thus supporting the involvement of the restoration of the immune response in the beneficial effects exerted by both CPM and gabapentin in the experimental model of IBS. Nevertheless, it is difficult to establish the association between immune activation and the development of symptoms in IBS patients [32]. However, different studies have revealed that a compromised epithelial barrier function, leading to abnormal intestinal permeability, correlates with low-grade immune activation and intestinal dysfunction in IBS patients [34,35,36]. This has been confirmed in the present study, since a reduced expression of the mucins *Muc-2* and *Muc-3*, two of the main proteins that constitute the mucus layer of the colonic mucosal surface, was observed in control IBS rats in comparison with the saline group (Figure 5). The altered mucin expression has been described in other experimental models of IBS in rats, like the water avoidance stress or the maternal deprivation [37,38]. The administration of CPM ameliorated the expression of both mucins; interestingly, the highest dose of CPM increased their expression to values significantly higher than those obtained in saline treated rats, thus reinforcing the barrier function in this experimental model of IBS. The impact of gabapentin on this marker of barrier function of the colonic mucosa was more modest than that showed by CPM, since it only significantly improved *Muc-3* expression, and this did not reach normal values (Figure 5A). Additional experiments revealed that CPM improved gut functionality by ameliorating intestinal permeability, which was assayed in vivo using FITC-dextran. FITC-dextran plasma levels in the control IBS rats were increased when compared to non-IBS control rats, which is in accordance with an altered epithelial barrier function observed in this experimental model of IBS. Of note, CPM treated rats reduced significantly FITC-dextran levels (*p* < 0.05; Figure 5B), whereas the reduction observed with gabapentin did not achieved statistical significance when compared with IBS control group (*p* > 0.05; Figure 5B).

The impaired barrier function in IBS seems to be closely related to an altered mucosal response to commensal intestinal bacteria, which may result in differential mucosal expression and responses of toll-like receptors, as evidenced both in human IBS and in experimental models [39,40]. In fact, in the present study, the colonic *Tlr-3* mRNA expression significantly increased in the DCA-control group in comparison with the saline-control group (Figure 5). This altered expression was only restored when DCA-treated rats were administered with the highest dose of CPM (Figure 4), thus revealing that the improvement in the barrier function may be associated with the down-regulation of the mucosal response to microbial contents in the intestinal lumen, avoiding different stimuli that can account for the visceral hypersensitivity that occurs in this experimental model of IBS. IBS is also associated with alterations in 5-HT homeostasis [41]. In this sense, the expression of the 5-HT receptor 4 (Htr-4) has been reported to be crucial in the pathogenesis of visceral hypersensitivity and the development of IBS [42]. When mRNA expression of Htr-4 was evaluated in this study, a six-fold increase was observed in the control group in comparison with the saline group (Figure 5, *p* < 0.05), which corroborates previous observations. However, none of the treatments modified the expression of this 5-HT receptor, which suggests that their mechanisms of action do not involve this pathway.

## 4. Conclusions

CPM improved the visceral hypersensitivity and the referred pain caused by DCA. The higher dose of CPM was as effective as the control drug gabapentin. Additionally, CPM enhanced the immune status of the animals reducing the expression of the pro-inflammatory mediators *Il1-β* and *Cox-2*, which could contribute to the visceral analgesia obtained, and restoring the intestinal epithelial barrier integrity by elevating the expression of the mucins *Muc-2* and *Muc-3*. The mechanism of action via the serotonin pathway seems unlikely to be involved since no effect was observed on the altered expression of the receptor *Htr-4*. Thus, it would be interesting to further study CPM as a new treatment for the prevention and/or treatment of IBS and also explore its capacity as a calcium source that could improve the altered calcium metabolism associated with this disorder.

## Figures and Tables

**Table 1:** Primer sequences used for real-time PCR assays.

Gene	Sequence 5′–3′	Annealing Temperature (°C)
***Gapdh***	FW: CCATCACCATCTTCCAGGAG RV: CCTGCTTCACCACCTTCTTG	60
***Il-1β***	FW: GATCTTTGAAGAAGAGCCCG RV: AACTATGTCCCGACCATTGC	59
***Muc-2***	FW: ACCACCATTACCACCACCTCAG RV: CGATCACCACCATTGCCACTG	60
***Muc-3***	FW: CACAAAGGCAAGAGTCCAGA RV: ACTGTCCTTGGTGCTGCTGAATG	60
***Cox-2***	FW: TGATGACTGCCCAACTCCCATG RV: AATGTTGAAGGTGTCCGGCAGC	60
***Tlr-3***	FW: GATTGGCAAGTTATTCGTC RV: GCGGAGGCTGTTGTAGG	60
***Htr-4***	FW: CAGTTGAAGTTGCCATCAGC RV: CGGCGAATTGGAGATGAACT	60
